# Toward the Assessment of Scientific and Public Health Impacts of the National Institute of Environmental Health Sciences Extramural Asthma Research Program Using Available Data

**DOI:** 10.1289/ehp.0800476

**Published:** 2009-03-24

**Authors:** Edward Liebow, Jerry Phelps, Bennett Van Houten, Shyanika Rose, Carlyn Orians, Jennifer Cohen, Philip Monroe, Christina H. Drew

**Affiliations:** 1 Battelle Health and Life Sciences, Seattle, Washington, USA; 2 Division of Extramural Research and Training, National Institute of Environmental Health Sciences, National Institutes of Health, Department of Health and Human Services, Research Triangle Park, North Carolina, USA; 3 University of Pittsburgh Medical Center, Pittsburgh, Pennsylvania, USA

**Keywords:** asthma, children, evaluation methodology, health impact analysis, minorities, policy, pulmonary organ systems/disease processes, susceptible populations

## Abstract

**Background:**

In the past 15 years, asthma prevalence has increased and is disproportionately distributed among children, minorities, and low-income persons. The National Institute of Environmental Health Sciences (NIEHS) Division of Extramural Research and Training developed a framework to measure the scientific and health impacts of its extramural asthma research to improve the scientific basis for reducing the health effects of asthma.

**Objectives:**

Here we apply the framework to characterize the NIEHS asthma portfolio’s impact in terms of publications, clinical applications of findings, community interventions, and technology developments.

**Methods:**

A logic model was tailored to inputs, outputs, and outcomes of the NIEHS asthma portfolio. Data from existing National Institutes of Health (NIH) databases are used, along with publicly available bibliometric data and structured elicitation of expert judgment.

**Results:**

NIEHS is the third largest source of asthma-related research grant funding within the NIH between 1975 and 2005, after the National Heart, Lung, and Blood Institute and the National Institute of Allergy and Infectious Diseases. Much of NIEHS-funded asthma research focuses on basic research, but results are often published in journals focused on clinical investigation, increasing the likelihood that the work is moved into practice along the “bench to bedside” continuum. NIEHS support has led to key breakthroughs in scientific research concerning susceptibility to asthma, environmental conditions that heighten asthma symptoms, and cellular mechanisms that may be involved in treating asthma.

**Conclusions:**

If gaps and limitations in publicly available data receive adequate attention, further linkages can be demonstrated between research activities and public health improvements. This logic model approach to research impact assessment demonstrates that it is possible to conceptualize program components, mine existing databases, and begin to show longer-term impacts of program results. The next challenges will be to modify current data structures, improve the linkages among relevant databases, incorporate as much electronically available data as possible, and determine how to improve the quality and health impact of the science that we support.

Growing demand for performance measurement and accountability in public research funding has led to a shift in focus for research impact assessment (RIA) beyond publications to measure accountability of research investment in broader terms, such as human health and environmental improvements as well as economic benefits. Conventional bibliometric analysis, which assesses contributions to knowledge, may not be sufficient to assess public health impacts.

Recent critiques of approaches to evaluating government-funded research underscore the need for better models and stronger linkages between evaluation and decisions about funding allocations (Coryn et al. 2007). Trochim and colleagues (2008), for example, propose an extensive mixed-methods approach that draws from diverse data sources to evaluate a large research initiative of the National Cancer Institute. A more tightly circumscribed approach is reflected in the work of Kuruvilla and colleagues (2006), who developed a research impact framework to help researchers systematically identify impacts resulting from their work. The tool also provides a structured framework for comparing research impacts across projects and time but is limited in that it includes data from only one source, the researcher. These researchers did not develop a full logic model, but rather identified specific descriptive categories within four broad areas: research-related impacts, policy impacts, service impacts, and societal impacts.

Increased interest in documenting the public health impacts from publicly funded research led the National Institute of Environmental Health Sciences (NIEHS) Division of Extramural Research (DERT) to develop a logic model to measure the impact of its extramural grant-funded investments in asthma research and to identify indicators to monitor changes over time (Figure 1). This more specific asthma-related framework was derived from work published earlier (Engel-Cox et al. 2008) to categorize the encompassing range of possible impacts of environmental health research related to human health, the economy, and environment. Presented here is a case study of how new methodologies and data sources can move us toward an assessment of public health research impacts, identifying data structure improvements needed to hasten this movement. The types of research impacts reported here were those judged to be feasible to assess related to the field of asthma. Our objective in the present study was to apply this conceptual framework in assessing the impacts of DERT’s extramural asthma research grants funded from 1975 to 2005.

We chose impacts of asthma research because of asthma’s public health significance. Asthma’s prevalence in the United States has nearly doubled since 1970, and it is now the most common chronic childhood disease, with a prevalence of 8.5% among children in 2004 (Moorman et al. 2007). In addition to morbidity and associated missed school days for children, asthma results in missed work days for parents, restrictions on physical activity, and other quality-of-life impairments. It is also well documented that persons from low-income and minority households are disproportionately affected by asthma (Brim et al. 2008; Persky et al. 2007) and that children from these homes are more likely to be treated in emergency and urgent care facilities because they lack adequate health care insurance coverage (Inkelas et al. 2008).

President Clinton’s 1997 Executive Order 13045 required each federal agency to identify and evaluate environmental health and safety risks that may hinder children’s health (Clinton 1997). The directive sparked an expansion of research into children’s health, including asthma, across the National Institutes of Health (NIH), the government’s principal agency for research that leads to improvements in public health. Within the NIH, NIEHS is the third largest source of asthma-related research grant funding [after the National Heart, Lung, and Blood Institute (NHLBI) and the National Institute of Allergy and Infectious Diseases (NIAID)]. From fiscal years 1975 to 2005, NIEHS awarded approximately $220 million in asthma-related grants, compared with approximately $1 billion and $324 million at NHLBI and NIAID, respectively, during the same time period (NIH Electronic Research Administration 2009).

NIEHS supports a multifaceted asthma research program, including basic research into asthma genetics, respiratory pathogenesis, and mechanisms by which particulate matter, aerosols, and aeroallergens induce asthmatic response; new methods for exposure measurement to dust and airborne allergens associated with asthma; national surveys to assess the risks of exposure to allergens and other environmental agents associated with asthma; the effectiveness of interventions that lower allergen levels in the home; and the effectiveness of interventions for secondary prevention of asthma.

## Approach

In 2006, DERT convened an expert panel to review the present state of knowledge about measuring the impact of research and to inform the development of the aforementioned conceptual framework that describes the agency’s extramural research portfolio (Engel-Cox et al. 2008). Panel members were selected based on their recognized research contributions along the whole bench-to-bedside-to-community translational continuum. In addition, we refined the conceptual model specifically for the asthma research portfolio and conducted a feasibility assessment of data sources and potential metrics of interest. Expert panel members were also asked to help assess the feasibility of accessing different data sources in conducting the evaluation and the validity of different metrics. We then compiled information from those data sources identified and developed attribution scenarios of how research impacts could be linked to the identified grant funding.

The conceptual framework uses a logic model format (Kellogg Foundation 2004). Components include contextual conditions, inputs, research activities, outputs, outcomes, and ultimate outcomes, with emphasis on science, clinical policy and practice, environmental policy and practice, and health outcomes. We developed indicators for each component using publicly available data sources (Appendix 1) and a structured elicitation of subject matter experts’ judgment.

In assessing the public health impacts of a research program, the most noticeable immediate outputs include the use of agency-supported research in subsequent research, such as citations in the scientific literature or in follow-up research studies. The Scientific Publication Information Retrieval and Evaluation System (SPIRES 2007) was used to link citations. Other possible immediate outcomes are the use of research in influencing the work of commissions, task forces, or advisory panels, and the dissemination of interventions beyond the initial research study. Potentially effective community-based interventions were linked to publications stemming from NIEHS-funded grant research. Appointees to these committees are often viewed as leaders in the field and as having influence on agency or science policy and guidelines. Composition of commissions, task forces, and agency advisory panels was reviewed to determine whether NIEHS-sponsored investigators participated. Immediate outcomes were assessed through bibliometric analysis, content analysis using the OmniViz content mapping and visualization software (Biowisdom, Inc., Cambridge, UK), and other database queries.

Intermediate outcomes result as immediate outcomes become translated into impacts. Three main types of impacts include economic, environmental, and health and social. Economic impacts may include changes in technology that affect asthma, such as new patents or processes, or new drugs developed. We searched the U.S. patent database and U.S. Food and Drug Administration (FDA) databases of drugs to compile this information. Environmental impacts may include changes in regulations, policies, or legislation that eventually result in changes in environmental measures, such as outdoor or indoor air quality indicators derived from U.S. Environmental Protection Agency (EPA) databases and U.S. federal legislative databases. Finally, health and social changes may include changes in guidelines (National Guideline Clearinghouse 2009) or health legislation (from U.S. federal and state legislative databases) that may eventually result in behavioral changes for health care providers, health systems, families, schools, or other youth settings. In a preliminary feasibility assessment (Liebow et al. 2006), we determined that regulations, legislation, and formal policy statements rarely cite the evidence on which they are based, so that such policy instruments cannot be attributed directly to specific research projects or centers. Impacts that can be more clearly attributed include economic impacts such as patents, technology changes, and drug development, as well as health and social impacts that include guidelines and practice changes.

Ultimate outcomes are a combined result of changes in intermediate outcomes over time and may include decreased asthma morbidity and mortality, incidence of asthma, emergency department utilization, decrease in hospitalization rates, use of rescue medicines, activity limitations among persons with asthma, and decrease in number of school or work days missed by persons because of asthma. These data were primarily compiled from asthma surveillance data available from the U.S. Centers for Disease Control and Prevention (CDC).

## Results

Results are summarized for each conceptual framework element: context, inputs, activities, outputs, and outcomes.

### Context

#### Annual congressional appropriations to NIH

Annual appropriations to NIH grew steadily from $4.3 billion in 1983 to $28.4 billion in 2005 (NIH Office of Budget 2008). During this same period, the NIEHS budget also grew, but at a slower pace, from $164.6 million to $720.2 million. The budgets of other NIH institutes and centers grew considerably faster, most notably at NIAID and NHLBI, growing from $278.9 million to nearly $4.3 billion and $624.1 million to $2.9 billion, respectively.

### Inputs

#### Grants and research funding

Between 1983 and 2006, DERT spent $218 million on 161 individual asthma-related research grants, adding to a total of 736 grant years [the aggregate number of years in which all individual principal investigators (PIs) affiliated with a specific institution received funding from NIEHS] (Table 1). Total extramural funding has increased over time, and asthma-related grant awards have risen as a percentage of the total extramural portfolio over time, climbing to a peak of 9.8% of the extramural portfolio in 2003 and dropping to 7.9% in 2005. The relative share of asthma-related funding invested in research has also increased over time.

#### Research mechanisms

NIEHS employs a range of grant mechanisms to fund asthma-related research. For the purposes of this analysis, mechanisms have been grouped into four general categories: training grants, innovation research/technology transfer grants, center grants (large multiproject grants issued to institutions), and research grants (Figure 2). Research grants are by far the most common type of award found in the asthma portfolio, but after 1999 the total funding for center grants (which include multiple coordinated projects as well as infrastructure grants) exceeds the funding for research grants. The number and funding of center grant awards fell between 2003 and 2005. The number of training grants related to asthma increased in the early 2000s, but the funds awarded for training grants are much lower, most likely because training grants provide only salary and tuition for predoctoral and postdoctoral trainees and do not support all research-related expenses. Innovation grants are the award type least commonly observed in the asthma portfolio.

#### Institutional profile

**Figure 1:**
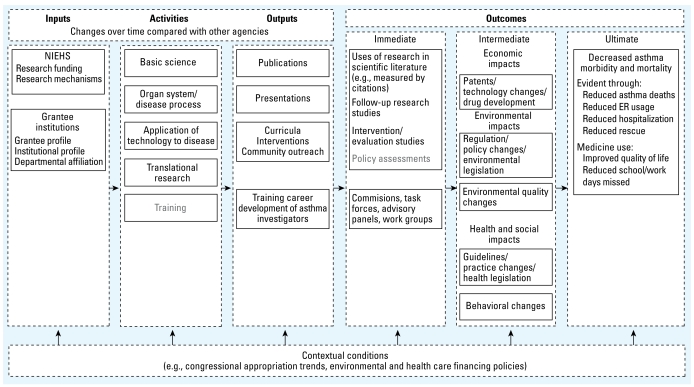
Conceptual framework for NIEHS asthma-related research portfolio. ER, emergency department. This logic model lays out inputs, activities, outputs, and outcomes of the NIEHS asthma-related research portfolio. Boxes represent types of information collected for the portfolio analysis. Gray text indicates gaps in electronically accessible data from which queries can be made and potential areas for future work.

Before 1996, the number of grants, PIs, and institutions funded were nearly identical. After 1996, the number of individuals and institutions funded for asthma research from NIEHS has mirrored the increases seen in the numbers of grants funded, but at lower rates of growth [see Supplemental Material, Figure 1 (available online at http://www.ehponline.org/members/2009/0800476/suppl.pdf)]. Over the 30-year study period, 76 different institutions received asthma-related NIEHS grants [see Supplemental Material, Table 1 (available online at http://www.ehponline.org/members/2009/0800476/suppl.pdf)]. Additionally, 63% of the funded institutions received funding for just one grant (although such grants may have continued over many years), and two institutions, Columbia University and Johns Hopkins University, received more than five unique asthma-related grants.

### Activities

**Figure 2:**
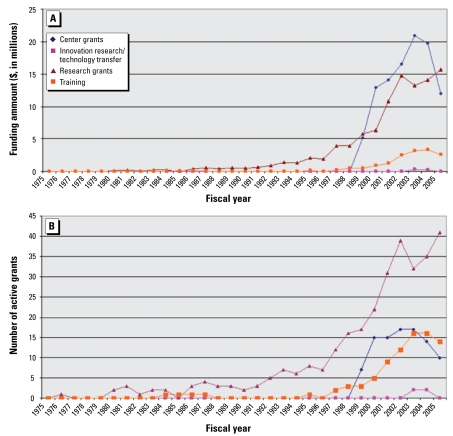
Funding amount (*A*) and number (*B*) of NIEHS asthma-related grants by research mechanism, 1975–2005. Research grants include P01, R01, R03, R15, R18, R21, R24, R25, R29, R33, R37, R55, R56, S (all), and U01 mechanisms. Center grants include G12, M01, P (all), U19, U42, and U54 mechanisms. Training grants include D (all), F (all), K (all), T (all), U2R, and U45 mechanisms. Innovation, research/technology transfer grants include R41, R42, R43, and R44 mechanisms.

The annual number and funding levels of active grants are classified according to six general activity categories: basic science (e.g., cellular/molecular, genetics); organ system/disease process (e.g., respiratory); application of technology to disease (e.g., Small Business Innovation Research/ Small Business Technology Transfer); center grants (e.g., Children’s Environmental Health Centers); training (e.g., fellowships); and translation (e.g., environmental justice, built environment) [see Supplemental Material, Figure 2 (available online at http://www.ehponline.org/members/2009/0800476/suppl.pdf)]. For both the number of active grants and amount of funds awarded over time, basic science research has steadily increased, with the rate of increase picking up beginning in 1998. A rapid increase in funds awarded for center grants occurred during 1998–2003 and then trailed off after 2003. Refinements in the activity classification scheme would benefit impact assessment, because the current categories are not mutually exclusive (each grant can be classified in up to two categories) and center grants often encompass multiple projects and centralized facility services associated with several types of activities either directly or indirectly.

### Outputs

#### Publications

The NIEHS asthma-related portfolio of 161 unique grants has 2,057 asthma-related journal publications associated with it (Table 2). Over the 30-year study period, the annual rate of publication from the asthma portfolio has roughly followed the portfolio’s funding profile, with a sharp spike in publications emerging after 1998. During the 1990s, the rate of publications increased before the dramatic funding increase, possibly indicating that preliminary studies accumulated to make a compelling case for increased research support. As funding increased dramatically, a time lag is evident, likely attributable to the time it takes to conduct environmental health research and publish findings in peer-reviewed literature. Even with the expansion of electronic publishing, most journals currently retain a dual print/electronic format, and page limits persist, leaving a time lag. The top 10 journals for NIEHS-supported research findings are a mix of basic science, clinical, and environmental health (Table 3). The total number of publications by a single PI ranged from 0 to > 100. Approximately 25% of PIs with grant awards were not associated with any publications, whereas approximately 7% of the NIEHS-supported asthma researchers were associated with grants generating > 100 publications. Approximately 75% of all the asthma-related publications acknowledging NIEHS support cited center grant awards.

#### Asthma-related interventions

A broad range of potentially effective community-based interventions were reported in publications stemming from NIEHS-funded grant research. Highlights of NIEHS-supported intervention research include such findings as control of asthma in children entails collaborative efforts and community-wide environmental control measures (Clark et al. 1999); neighborhood asthma coalitions are associated with promising reductions in acute care rates among active program participants (Fisher et al. 2004); and intensive cleaning can produce significant reductions in cockroach allergen in homes with heavy initial cockroach infestations (McConnell et al. 2003).

Overall, evidence of outputs is widely available in publicly available data sources directly traceable to specific grants. These linkages have been possible only recently with the development of the SPIRES application (developed in large part at NIEHS and now available across NIH) that finds peer-reviewed articles in the PubMed database (http://www.ncbi.nlm.nih.gov/sites/entrez?db=pubmed) that cite NIH publications. There are some limitations in these data, however. Despite a recent congressional mandate (NIH Reauthorization Act of 2006), grantees do not always include grant numbers in publications, important journals are not included in the PubMed database, and older grant numbers are not always unique. These challenges are expected to improve over time, and we are reasonably confident that the outputs attributable to the asthma portfolio are representative of the majority of papers produced.

### Immediate outcomes

#### Citations

The 2,057 publications attributed to the NIEHS asthma-related portfolio have been cited a total of 29,638 times (1975–2005), with a median of 2.1 citations for each publication (mean ± SD, 14.1 ± 12.9). About 82% of the publications are later cited by other articles. As shown in Table 3, the publications that cite NIEHS-sponsored work are consistently cited in journals with impact factors of 1.9–8.7. Of the articles later cited, the average length of time until first citation was less than 1 year (first cited in the same year published), and > 98% of all articles are cited within 3 years.

#### Publication content

**Figure 3:**
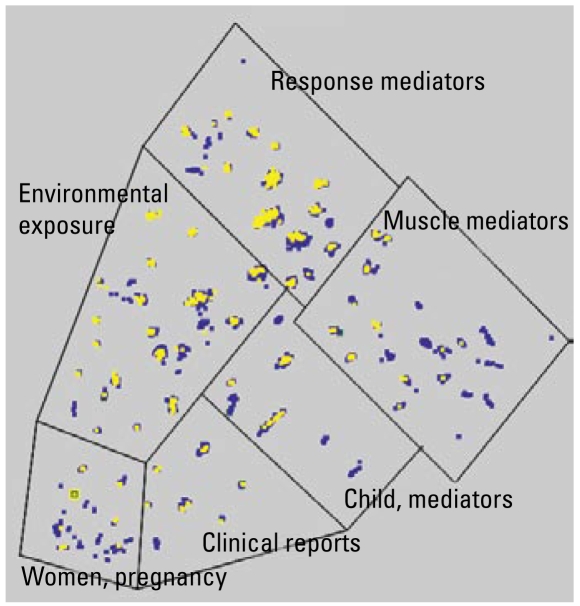
Asthma-related publications from NIEHS and comparison agency-sponsored research. Taking the abstracts of all asthma-related publications resulting from the NIEHS and comparison agency grant awards (1975–2005), the OmniViz program was used to assess word occurrence, distribution, and associations in the title and abstract of each publication to define the major discriminating themes and to cluster documents with related themes. Proximity is a measure of thematic similarity. The closer the papers are, the more similar they are. Six clusters emerge, each outlined in a box. The blue nodes indicate publications sponsored by the comparison agencies, whereas the yellow nodes indicate publications sponsored by NIEHS. Substantively, NIEHS has concentrated more of its efforts in the response mediators and environmental exposures domains.

**Figure 4:**
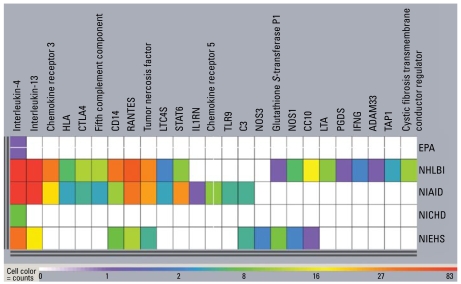
Distribution of genes of interest across grants, by agency. This CoMet visualization highlights the specific genetic foci (columns) associated with each agency’s asthma-related research portfolio (rows). The warmer the color (i.e., yellow, orange, and red), the greater the number of grants awarded for research on a particular genetic location or complex. The cooler the color (green, blue, purple), the fewer the number of grants awarded for a particular genetic location or complex. This visualization shows the varied agency interests in specific genes, indicating unique niches and areas of overlap. NIEHS, although not awarding as many grants as its NHLBI and NIAID counterparts, is focusing its resources in a manner mostly consistent with these two institutes. A gene that is hot in NIEHS-supported grants but studied at only one other institute is glutathione *S*-transferase, an oxidative stress gene often associated with environmental exposures such as ozone.

Comparative content analysis of asthma-related publications generated from NIEHS and other agencies revealed distinctive patterns relating to the balance of clinical and preclinical work, genetic susceptibility, and environmental influences of asthma. These analyses use the OmniViz application, which has tools to assess word occurrence, distribution, and associations to define major discriminating themes and to cluster documents with related themes. Such themes may be more general (e.g., hyperreactivity) or involve more complex Boolean constructs (e.g., hyperreactivity AND eosinophil NOT parasites). Publications were clustered into areas of similarity by word analysis of title and abstract. The results of the cluster analysis are visualized for our present purposes in a galaxy diagram (Figure 3) in which cluster proximity reflects semantic relatedness. Fittingly, NIEHS research (indicated in yellow) is clustered most heavily in the environmental exposure and response mediators areas. Another visualization tool, the CoMet diagram (OmniViz) (Figure 4) captures a global assessment of relationships between specific genetic research and sponsoring agencies, providing a graphic way to rapidly focus on points of interest. Work since 1996 has associated 79 genes with asthma phenotypes, seven of which have been associated with asthma phenotypes in five or more studies (Ober and Hoffjan 2006). NIEHS has funded research on genes associated with asthma susceptibility and has focused funding of genetic research on interleukin-4 and interleukin-13, two cytokines that, among other things, help regulate adaptive immunity to allergens and other asthma triggers. This type of visualization helps distinguish the various niches for each of the funding agencies.

#### Role of the environment in asthma susceptibility

NIEHS also has a large concentration of asthma-related publications concerning environmental toxicants on the National Priority (Superfund) List (U.S. EPA 2008). Research focusing on the chemicals identified as the most hazardous among known or threatened releases has significant potential for public health improvement. Toxicants that are mentioned prominently in publications from NIEHS-sponsored research include air pollution ingredients (e.g., carbon monoxide and other products of combustion; polycyclic aromatic hydrocarbons, including naphthalene and other benzene compounds), environmental tobacco smoke and its constituent ingredients, agricultural chemicals, and lipopolysaccharides and other endotoxins.

#### Commissions, task forces, advisory panels, work groups

Development of expertise that then informs health policy changes is one public health outcome of interest to DERT. The 2005 membership of various expert groups commissioned to assess the need for policy change was cross-referenced with the roster of NIEHS asthma grant awardees. We identified multiple FDA and Institute of Medicine (IOM) commissions, task forces, advisory panels, and workgroups relevant to asthma. For example, recent FDA science advisory panels include the Anesthesiology and Respiratory Therapy Devices Panel and the Pulmonary-Allergy Drugs Advisory Committee. For IOM, groups examined included current IOM members, as well as more specialized groups such as the Environmental Health Sciences, Research, and Medicine Roundtable. We also identified a current advisory panel convened by the Joint Commission on the Accreditation of Healthcare Organizations (The Joint Commission) to recommend asthma-related hospital performance measures. In addition, we included advisory panels convened to develop the two major clinical guidelines for asthma care: The Global Initiative for Asthma and the National Asthma Education and Prevention Program (NAEPP). Ten NIEHS agency-funded PIs could be identified as current participants in IOM, Joint Commission, and NAEPP panels.

### Intermediate outcomes

#### Economic impacts

Asthma can be a disabling chronic disease that results in lost work and school days, emergency department visits, and other adverse impacts with significant financial dimensions. Thus, research that leads to improvements in disease management and reducing the incidence of asthma can have substantial long-term economic payoff. However, there is also an intermediate sense in which research funding can result in the development of devices and disease management techniques that have an economic benefit for organizations that have development successes. Patents provide one concrete, albeit limited, indicator for intermediate economic impacts of research funding.

Research resulting in patents is not always traceable to the funding organization (Anderson et al. 1996, Narin 1994), because the findings may be cited as “prior art” and only indirectly attributable to the funding organization. A search of the U.S. Patent Office online database (all patents since 1790) and focusing on the Government Interest field yielded 66 patents with ES listed as a part of a grant number. Of these, four patents (all to one PI) matched to a specific NIEHS-funded grant. Emerging efforts at NIEHS and NIH are working to improve data structures and to link patent data with grant funding.

#### Technology development

Drug development is based on clinical trials research, supported by basic research in biochemistry and pathogenesis, and followed by postmarketing surveillance and clinical case reports. Clinical trials are often sponsored by pharmaceutical companies, although NIH has reaffirmed its commitment to support clinical research over the past decade (Zerhouni 2003). We searched FDA drug approval dockets for publications cited that acknowledge NIEHS sponsorship, and identified 39 known asthma medications. Information about the new drug applications for each of the drugs was searched on the FDA Web site. Bibliographic references for each drug were compiled and then matched against the master bibliographic database associated with asthma-related grants. NIEHS-sponsored research results in publications concerning asthma drugs cromolyn, albuterol, epinephrine, and fluticasone. Although the association is somewhat weak, we were able to link NIEHS research to drug research. Many might argue whether this is appropriate, as the main mission of NIEHS is to study the environmental factors contributing to susceptibility rather than to develop therapeutic treatments. However, because many of the mechanistic processes are related, it is natural that there would be at least some overlap.

#### Additional impacts and attribution difficulties

Ideally, the NIEHS asthma program logic model (Figure 1) would contain direct links between intermediate outcomes to additional impacts such as

- Environmental impacts—changes in legislation and regulations leading, in turn, to improvements in environmental quality.
- Health and social impacts changes in health legislation, reductions in asthma mortality, emergency department utilization, hospitalization rates, rescue medicine use, and improvements in quality of life as indicated by surveillance measures such as the CDC’s Behavioral Risk Factor Surveillance System, school and work days missed.

Indicators for all of these impacts have been included in a monitoring and evaluation database that NIEHS has developed for periodic research impact assessment (unpublished database). However, in none of these cases were we able to link trends to specific research activities because either *a*) it was not feasible to collect the data, or *b*) the data sources available had no connection to publications or grants to make such a linkage. For example, legislative databases often include a legislative history and expert testimony in support of legislation, but in the most optimistic scenario, this background information takes the form of “research has shown that . . .” without actually citing any specific researcher’s work. It is difficult to obtain information without interviewing individuals about the role of science funded by NIEHS in developing legislation or regulation. Future assessments will address these issues with some of the end users of such research, that is, regulators and legislators.

## Limitations

We have assumed from the outset that to be transparent and sustainable, periodic evaluations of the research portfolio and its public health impacts should be based on indicator data that are

- Readily accessible to NIEHS staff
- Consistently organized over the assessment period
- Electronically available
- Documented with respect to population covered, geographic area covered, and interval or frequency of collection.

Through this initial effort we have discovered that indicator data meeting these criteria are generally available to support conventional bibliometric analyses of research publications. Certain limitations apply, however. Beyond publications, indicators of other activities, outputs, and outcomes are not as well supported. Refinements to current data structures within the agency, such as estimating the portion of a given center grant related to a disease, condition, or health end point, would assist with public health impact assessments. Conference papers and presentations, often a channel for early release of findings, rarely include publicly available information about funding sources. Commission/taskforce membership is difficult to cross-reference with grant recipients because there are no central repositories of active group rosters for comparison with author lists. In addition, although current members of such groups are often listed on agency Web sites, past commission/task force membership lists are not easily available. Thus, it is difficult to know whether agency-sponsored PIs participated in such activities in the past. Legislation, which can have a profound impact on public health, almost never cites research findings that may well be what has prompted the legal intervention. Additional work is underway to survey asthma researchers funded by NIEHS (1975–2005) and likely end users of this research to obtain their views on dissemination channels most likely to have policy and public health impacts.

Additionally, grant data available in the Information for Management, Planning, Analysis, and Coordination (IMPAC II) database at the time of this analysis were restricted to title and abstract, thus limiting the extent of information that could be extracted. For example, additional information about associated genes is likely available in the “specific aims” sections of grant applications but until recently were not searchable. Similarly, annual progress reports, which are not searchable within the IMPAC II database, contain a wealth of information about the actual research conducted (not just the proposed research) and about results and outcomes that may not be publishable findings and thus might not emerge in a bibliometric analysis.

The ultimate outcome measures, representing indicators of decreased asthma morbidity and mortality, are also limited. Ultimate outcomes take the longest time to realize and may not be observed for ≥ 10 years after research findings are first reported. Although ultimate outcome measures should be included in our long-term assessments of scientific and public health impacts, they are the least direct of all, and attribution to a particular research project or program will likely never be definitive.

## Conclusions

Although conventional bibliometric methods can be useful to understand improvements in knowledge relating to public health advances, these methods alone are insufficient to assess the broad range of contributions made possible by research funding. Instead, using a conceptual framework and reviewing a broad variety of existing electronic databases can help to shed additional light on the types of outcomes that are possible and salient for assessing research impact. NIEHS has taken a significant step toward implementing a framework that helps us think globally about specific mechanisms by which research investments contribute to public health improvements. Although the nature of scientific discovery is well enough infused with contingency and serendipity to thwart any effort to achieve definitive attribution of public health improvements to a specific research grant award, a portfolio analysis like the one presented here clearly points to more immediate outcomes that, in the aggregate, become the necessary and sufficient conditions under which such public health improvements are achieved.

The call for increased transparency and accountability in federal science policy requires that we think strategically about modifications to current data structures that allow us to track expenditures and research results over time. Improved linkages between relevant databases (such as searchable patent and investigational new drug applications with the federal grant database) will assist with attribution of near-term and intermediate outcomes to research funding. Improved data structures and the ability to search progress reports and specific aims side by side with grant titles and abstracts will increase access to scientific output data useful for impact assessment. As the use of publication data increases, grantees will become even more aware of the importance of acknowledging grant support, creating a more complete picture of the publication products of the extramural research portfolio. This in turn may increase the likelihood that grant support will be properly acknowledged in publications and downstream clinical and public health applications.

Making sense of the vast and highly segmented landscape of research reports and publications produced with public grant support is a challenge, but new content analysis tools, coupled with visualization techniques, show great promise in taking documents in diverse formats to highlight patterns over time and across narrowly defined subspecialty areas.

NIEHS efforts reported here and in the companion article by Engel-Cox et al. (2008) demonstrate that it is possible to conceptualize program components, mine existing databases, and begin to show longer-term impacts of program results. The next challenges will be to modify current data structures, improve the linkages among relevant databases, and determine how to improve the science that we support. DERT’s charge is to distribute resources in ways that produce the best science to achieve a healthier population. We can now begin to ask questions such as: How should DERT structure its funding portfolio to create the best chances to produce innovation? How should investigators be made aware of ways in which their work can inform policies and environmental management regimes that, in turn, lead to public health improvements? To create interdisciplinary synergies, what is the ideal portfolio blend of institutional depth and breadth of research investments? We cannot answer these questions now, but the framework allows us to sharpen the focus of our thinking about how to structure information to answer these questions in the future.

## Figures and Tables

**Table 1:** NIEHS asthma-related grants compared with total extramural grants, 1983–2005.

Fiscal year	Total no. of NIEHS extramural grants	Total no. of active NIEHS asthma-related grants	Total NIEHS extramural funding ($)	Total NIEHS extramural asthma-related funding ($)	Total extramural asthma-related (%)
1983	821	4	60,736,000	310,635	0.51
1984	834	4	68,882,000	314,222	0.46
1985	876	1	78,523,000	59,011	0.08
1986	839	4	79,238,000	505,059	0.64
1987	869	5	89,803,000	653,824	0.73
1988	894	3	90,198,000	413,336	0.46
1989	855	3	92,923,000	603,383	0.65
1990	896	2	97,001,000	473,105	0.49
1991	1,000	3	103,996,000	646,597	0.62
1992	929	5	113,469,000	938,711	0.83
1993	909	8	115,708,000	1,447,726	1.25
1994	966	8	129,238,000	1,395,976	1.08
1995	943	10	130,212,000	2,231,820	1.71
1996	1,021	7	139,670,000	1,908,528	1.37
1997	996	16	149,399,000	4,253,311	2.85
1998	1,036	21	159,321,000	4,428,412	2.78
1999	1,072	29	187,196,000	11,526,989	6.16
2000	1,144	81	224,091,000	20,169,948	9.00
2001	1,264	99	318,519,000	26,190,674	8.22
2002	1,308	124	368,815,000	33,802,154	9.17
2003	1,305	96	385,440,000	37,742,742	9.79
2004	1,288	103	383,233,000	37,368,968	9.75
2005	1,237	100	383,925,000	30,476,463	7.94
Total	23,302	736	3,949,536,000	217,861,594	5.51

**Table 2:** Top 10 journals publishing asthma-related research articles funded by NIEHS.

Journals	No. of publications (%)
All NIEHS-funded publications	2,057 (100)
*Environmental Health Perspectives*	205 (10)
*American Journal of Respiratory and Critical Care Medicine*	74 (4)
*Toxicology and Applied Pharmacology*	60 (3)
*Journal of Allergy and Clinical Immunology*	51 (2)
*Journal of Biological Chemistry*	48 (2)
*American Journal of Respiratory Cell and Molecular Biology*	47 (2)
*American Journal of Physiology*	41 (2)
*American Journal of Physiology-Lung Cellular and Molecular Physiology*	40 (2)
*Journal of Applied Physiology*	39 (2)
*Cancer Epidemiology Biomarkers and Prevention*	35 (2)

**Table 3:** Top 10 journals containing citations to NIEHS grant-related publications, by citation count, research level, and subfield.

Journal (citation count)	2005 Journal impact factor	Journal research level^a^	Journal subfield^a^
*Environmental Health Perspectives* (537)	5.34	Clinical investigation	Clinical medicine/environmental and occupational health
*American Journal of Respiratory and Critical Care Medicine* (412)	8.69	Clinical mix	Clinical medicine/respiratory system
*Toxicological Sciences* (302)	3.09	Clinical investigation	Clinical medicine/pharmacology
*Toxicology and Applied Pharmacology* (297)	3.15	Clinical investigation	Clinical medicine/pharmacology
*American Journal of Physiology– Lung Cellular and Molecular Physiology* (291)	3.94	Basic	Biomedical research/physiology
*Inhalation Toxicology* (269)	1.89	Clinical investigation	Clinical medicine/pharmacology
*Journal of Biological Chemistry* (260)	5.85	Basic	Biomedical research/biochemistry and molecular biology
*Journal of Allergy and Clinical Immunology* (256)	7.67	Clinical mix	Clinical medicine/allergy
*American Journal of Respiratory Cell* a*nd Molecular Biology* (244)	3.99	Basic	Biomedical research/biochemistry and molecular biology
*European Respiratory Journal* (239)	3.95	Clinical mix	Clinical medicine/respiratory system

a Data from ipIQ Corporation, 2005. CI/SSCI Journal Classification File, prepared by ipIQ for the National Science Foundation under NSF contract SRS0002731, 19 August 2005 (unpublished data).

**Appendix 1:** Summary of data sources for Asthma Portfolio Research Impact Assessment.

Data category	Sources
Input indicators	

NIH budget data	NIH Office of the Budget (http://officeofbudget.od.nih.gov/UI/history.htm)
NIH asthma-related budget data	IMPAC II
	NIEHS internal analysis of NIEHS center grants
Non-NIH agency budget data	IMPAC II [http://era.nih.gov/impacii/index.cfm (for authorized users only)]

Activity indicators	

Grant awards, institutions, PIs	IMPAC II
	U.S. EPA, National Center for Environmental Research (http://es.epa.gov/ncer/grants/)

Output indicators	

Publications	SPIRES
	PubMed (www.pubmed.gov)
	U.S. EPA (http://es.epa.gov/ncer/grants/)
Curricula, interventions, and outreach materials	NIEHS (http://www-apps.niehs.nih.gov/outreach-education/)
	U.S. EPA (http://www.epa.gov/iaq/schools/asthma/)
	(http://www.epa.gov/asthma/)
	(http://www.epa.gov/asthma/community.html)
	(http://www.epa.gov/asthma/publications.html)
	CDC (http://www.cdc.gov/asthma/links.htm)
	(http://www.cdc.gov/asthma/default.htm)
	NHLBI (http://www.nhlbi.nih.gov/health/public/lung/index.htm#asthma)
	NAEPP (http://www.nhlbi.nih.gov/about/naepp/index.htm)
	ATSDR (http://www.atsdr.cdc.gov/HEC/CSEM/asthma/index.html)
	NIOSH (http://www.cdc.gov/niosh/topics/asthma/#prevention)
	NIAID (http://www.niaid.nih.gov/publications/asthma.htm)
	NLM (http://www.nlm.nih.gov/medlineplus/asthmainchildren.html)
	American Lung Association (http://www.lungusa.org/site/pp.asp?c=dvLUK9O0E&b=22542)
	American Academy of Allergy, Asthma and Immunology (http://www.aaaai.org/)
	CDC Asthma Intervention Database (http://www.cdc.gov/asthma/interventions/default.htm)

Outcome indicators	

Citation database	Thompson Scientific Institute for Scientific Information Science Citation index and journal citation reports (http://www.thomsonreuters.com/products_services/scientific/Journal_Citation_Reports)
Commissions, task forces, advisory panels, work groups	Institute of Medicine (http://www.iom.edu/)
	FDA science advisory panels
	Agency for Healthcare Research and Quality (http://guidelines.gov)
Patents	U.S. Patent Database (www.uspto.gov/patft/index.html)
Drugs	FDA’s Center for Drug Evaluation and Research (CDER) (http://www.accessdata.fda.gov/scripts/cder/drugsatfda/)
Legislation	Federal (http://thomas.loc.gov)
	State–National Conference of State Legislatures:
	Asthma-Related State Legislation and Statutes Database (http://www.ncsl.org/default.aspx?tabid=17322)
	Air Quality Policy Database (http://www.ncsl.org/programs/environ/air/airqualitydb.cfm)
Guidelines and care standards	National Guidelines Clearinghouse Database (http://www.guidelines.gov/)
Environmental changes related to air quality	U.S. EPA Air Quality System Database (http://www.epa.gov/air/data/aqsdb.html)
Asthma mortality	National Center for Health Statistics National Vital Statistics System (http://www.cdc.gov/mmwr/preview/mmwrhtml/00052262.htm#00003083.htm)
Emergency department utilization	National Center for Health Statistics National Ambulatory Medical Care Survey (http://www.cdc.gov/mmwr/preview/mmwrhtml/ss5101a1.htm)
Hospitalization	National Ambulatory Medical Care Survey (http://www.cdc.gov/mmwr/preview/mmwrhtml/ss5101a1.htm)
Rescue medicine use	CDC’s National Asthma Survey (http://www.cdc.gov/nchs/about/major/slaits/nas.htm)
Quality of life indicators	CDC’s Behavioral Risk Factor Surveillance System
	National Center for Health Statistics National Health Interview Survey (http://www.cdc.gov/mmwr/preview/mmwrhtml/ss5101a1.htm#tab3)
